# Guy's Hospital Reports

**Published:** 1841-10

**Authors:** 


### Art. II.

*Guy's Hospital Reports.* Nos. X-XI-XII. *April and October*, 1840, *and April*, 1841.—*London.* 8vo.

These excellent Reports hold a steady course of usefulness, yielding at convenient intervals much valuable information on a great variety of topics. Some gleanings from the three numbers now before us are here offered to our readers.

No. X.

*Some Observations tending to demonstrate the Dependence of Vascular Organization upon Physical Causes.* By Sir Anthony Carlisle, F.R.S.—This is a paper of thirteen pages, containing several very curious observations and experiments, illustrated by diagrams. It is well worthy of perusal; but being of a nature which does not admit of abbreviation, our curious readers must themselves look to the original.

*Observations on the Existence of certain Elements of the Milk in the Urine during Utero-gestation; and on the Application of this fact to the Diagnosis of Pregnancy.* By Golding Bird, M.D. F.L.S.—Dr. Bird has in this paper communicated the result of some experiments made by him on that new constituent supposed to exist in the urine of pregnant women, termed *kiestein.* When the urine is allowed to repose in a cylindrical vessel for a few days, this substance appears, first, in the form of a cottony cloud, which shortly rises to the surface, forming, there, a fat-like scum, and sinks again in minute flocculi to the bottom of the vessel. This crust of kiestein is stated to be distinguishable from analogous pellicles which occasionally form on the surface of the urine, from its never becoming mouldy or remaining on the surface beyond three or four days from the time of its complete formation. The discovery of it is considered important as aiding the diagnosis of pregnancy; and Dr. Bird has experimented both on pregnant women and others presumed not to be so with reference to this application of it. Of thirty pregnant women, the urine in all but three gave out copious fat-like pellicles after two or three days' exposure, and the exceptions Dr. Bird accounts for by the fact, that in them there existed, at the time of making the experiments, considerable febrile excitement. Of a number of unmarried females experimented on at the same time and in a like manner, in two instances only was any evidence of the presence of this peculiar matter manifested; and in both these, though they indignantly repudiated the imputation of pregnancy at the time, yet such afterwards turned out to have been the fact. Strong presumptive evidence is derived from these statements if they are free from the possibility of error. In one of the last-named experiments the urine was exposed in a lightly-covered glass cylinder: in two days a dense pellicle of fat-like matter formed on its surface; this increased in thickness during three days, and then evolved a powerful odour of putrefying cheese. From Dr. Bird's investigation of the chemical nature of this kiestein, he considers that

"The greasy aspect of the pellicle arises, not from the presence of fat, but from the numerous crystals of triple phosphate, which, from their brilliancy, produce this glistening appearance; with regard to the nature of the animal matter mixed with the crystals, it is difficult, in the present state of physiological chemistry, to give a positive opinion; it is not mere albumen or casein, although much closer allied to the latter than to any other product of organization I am acquainted with, especially when we connect with its chemical character the powerful cheese-like odour so frequently evolved during its development in the urine, in the form of a pellicle...........The crust of earthy phosphate which forms on the surface of all urine by long repose, cannot be mistaken for the pellicle under consideration, as that which destroys the latter, viz. putrefaction, causes the production of the former."

Finding evidence of the presence of certain ingredients of milk, as caseous matter and earthy phosphates, in the urine of pregnant women, Dr. Bird suggests as a probable explanation, that during uterine gestation these ingredients are eliminated from the blood in the breasts, and not finding an exit therefrom, are taken up thence and carried to the kidneys, where they are discharged with the urine. According to Dr. Bird's experience, and a few striking cases in point are detailed, such pellicles are not formed in the urine after parturition, when the milk with its caseous elements finds a ready discharge from the nipple.

As a test of the existence of pregnancy, the formation of the caseous pellicle, especially if accompanied with a cheese-like odour, will, Dr. Bird has no doubt, be a valuable corroborative indication. Connected with other symptoms he would regard it in that light; but if existing alone and unsupported by other indications of pregnancy, we have no right, he thinks, in the present state of our knowledge, to regard it as conclusive. The following are the deductions of the author:

"1. That certain organic matters, closely resembling, if not identical with caseous matter mixed with abundance of the earthy phosphates in a crystallized state, are eliminated from the blood during pregnancy; and, if not otherwise removed, are taken up and finally thrown out of the system by the kidneys.

"2. That certain accidental circumstances, especially connected with those morbid actions in which the kidney is called upon to perform a compensating function for the skin, as indicated by the abundance of azotized matter in the form of amorphous lithate of ammonia in the urine, interfere temporarily with the development of caseous matter, as they do in checking the cutaneous and other secretions.

"3. That, taken in connexion with other symptoms, as the formation of a dark areola round the nipple, and cessation of menstruation and abdominal enlargement, the formation of a caseous pellicle in the urine affords a very valuable corroborative indication of the existence of pregnancy."

The two next papers are by T. Wilkinson King, Esq.; one *On the Common Action of the Auriculo-ventricular Valves;* the other *On the Surfaces of Contact, or Attrition, on the Valves of the Heart.* (With Plates). But as there is little in them that is not already known, and as even the author himself yields assent to their want of originality, we are relieved from the necessity of noticing them further.

The next is a long and elaborate paper giving *An Account of the Fibrous Structure of the Subserous Membrane of the Aorta;* by Norman Chevers, m.d., which, likewise, we need not do more than refer to, as we find that without the plates which accompany the paper we would fail in making it intelligible. Indeed, at all events, as we are gleaning from hospital reports, we prefer, in this place, keeping to our text.

The paper which follows is one of much practical value, and deserves a more lengthened notice than we can bestow on it. It is *On the Morbid Consequences of Undue Lactation.* By Samuel Ashwell, m.d.—But little has been written on this subject; Dr. Marshall Hall being the only author who has bestowed on it more than a few incidental remarks. Dr. Ashwell considers exhaustion to be the permanent morbid state associated with undue suckling; and the following circumstances regarding the affection may, he thinks, be proved: *First.* That lactation to be morbid need not be long; evil consequences may ensue soon after its commencement; occasionally within a few weeks; more frequently within a period protracted beyond nine months. *Secondly.* That organic lesions may, though rarely, result from undue suckling. *Thirdly.* That weaning the child is generally indispensable to the cure—the remedy without which all others will be inefficient.

It would be injustice to curtail the already succinct but clear account of the history and symptoms of undue lactation; and our prescribed limits do not permit us to give them in full: we particularly recommend them to the notice of junior practitioners.

*Functional Amaurosis,* accompanied by congestion of the conjunctiva, are frequent results of excessive lactation; as are also specks and slight ulcerations of the cornea. In these cases the sufferer may be encouraged to expect the restoration of sight provided there be no serious organic change. Weaning, avoidance of quickly-recurring pregnancy, improved diet, repeated small blisters near the eye, are the proper remedial means. *Jactitation* is an occasional attendant on this state. In the cases reported by Dr. Ashwell, leucorrhœa and general debility were present. *Epilepsy* is sometimes a product of over-suckling, on the same grounds as inanition, losses of blood, and deficiencies in its quantity and quality are productive of this malady. *Insanity*, more or less permanent, may originate from over-lactation; the progress and symptoms of such affection are well told by Dr. Ashwell. The *pathology* of these functional results of undue suckling is by no means intricate or unsatisfactory. An impaired and attenuated condition of the blood, and a consequently depressed state of the nervous system, especially of the organic system of nerves, is the clue by which all the symptoms may be unravelled. Cases are given by Dr. Ashwell wherein organic changes in the brain, lungs, and uterus were induced by very prolonged undue suckling. Respecting headach we are presented with the following sensible observations:

"It is a frequent concomitant of the malady; nor can the practitioner be too strongly impressed with the hazard arising from its continuance. So long as it is general, not very severe, and transient; so long as it does not recur periodically, with marked premonitory symptoms, it may be viewed as comparatively free from risk. But, if it be dreaded on account of the permanent uneasiness which it has already produced, or from its intensity and acuteness; if it seize on one part of the head and remain fixed there; if its paroxysms be preceded by rigors, and if the pain never entirely subsides; more especially if there be partial paralysis, mental peculiarity, or forgetfulness approaching to imbecility, or any other anomalous symptom indicative of deranged nervous action, for instance, an unusual affection of the eye, such as double or impaired vision; or of the auditory nerve injuring the hearing, or rendering it excessively and painfully acute; or if there be impeded deglutition, then danger exists, and a softened or otherwise structurally altered condition of the brain may be feared.

"*Treatment.* The indications in the merely functional affections are not difficult to meet. Where the symptoms of exhaustion are slight, a better diet, a careful regulation of the bowels, a tonic treatment, and, above all, diminished suckling, will often avail. Nor is it necessary to urge very strongly, because the propriety of the recommendation is evident, that the child should be fed two or three times within the twenty-four hours, and that unbroken sleep during the night should be secured to the mother. But let it be remembered that this will not always avail. A continuance of the debility, or aggravated prevalence of one or more of the symptoms already enumerated, will plainly indicate the necessity of entire weaning. If the child be purged, or become gradually emaciated, it will corroborate the importance of the step. Where organic disease is threatened, especial attention must be paid to the organ in which it seems likely to occur. Cupping or leeches may be required; and counter-irritation by blisters, setons, or issues may be expedient; beyond these general directions, the practitioner will proceed according to the exigencies of the case, never omitting the weaning of the child. The convalescence of such patients is generally protracted and difficult, years sometimes elapsing prior to recovery. Nor can it be too forcibly recommended that suckling should be abandoned if a fresh pregnancy succeed very quickly. The symptoms are often rendered worse by gestation and invariably by lactation. Iron, chalybeate waters, country and sea air, travelling, and exercise are most important auxiliaries."

*Practical Hints on the Treatment of Stricture.* By Bransby Cooper, f.r.s.—This is a valuable communication, which, as well as a second part in the next number of the Reports, we shall notice elsewhere. We recommend the perusal of this memoir to all who are addicted to the heroic treatment of stricture; they cannot fail to profit by its perusal.

*Cases of Excision of the Elbow-joint.* By C. Aston Key, Esq.—Mr. Key has lately performed two operations of this kind on men, one twenty-six, the other forty-three years old. They were both cases in which, to save life, amputation must have been practised had not excision been had recourse to. The first left the hospital with some motion in the joint, and with considerable power in the limb, and shortly after returned to his occupation as a postman; the second, a shipwright, was even more fortunate, as his convalescence was both more rapid and more complete.

January 14, 1840. *Operation* in case ii. "An incision was made posteriorly in either side of the joint, about four inches long; another transversely, and the flaps reflected; the articular extremity of the humerus was then sawn off, also the olecranon and coronoid process of the ulna; the head of the radius was left; two or three vessels required ligature; about a pint of blood was lost. The divided edges were brought together by sutures and plaster; the arm was then laid on a splint. The operation lasted twenty minutes, and the patient bore it remarkably well." Nothing untoward occurred subsequently, and on the 20th February the following note was taken. "From the last date he has been recovering his strength rapidly; wound quite healed; can flex the arm to a right angle, and extend it to an obtuse one with ease and comfort; has considerable strength in the arm, as he can raise a heavy stool above his head; feels confident of being able to follow his employment, which is a very laborious one." On examination of the portions of bone excised, the cartilaginous surfaces were found completely destroyed by ulceration.

The improvement that ensues after the operation shows how little the constitution suffers from it, severe as it appears during its performance. The nature of the parts excised explains the little constitutional disturbance; no veins or arteries of magnitude being compromised in the operation, and the freedom of the incisions favouring the exit of the discharge that attends the healing of the wound. The removal of a source of irritation seems to be immediately felt in the improved appearance, refreshing sleep, and returning appetite, aided, no doubt, by a suppurating cavity with an imperfect outlet being converted into an open suppurating wound. Mr. Key considers that the most favorable cases for such an operation are those in which the disease has had its beginning in the articular surfaces; and that on this account the operation will be likely to succeed better in adults than in young subjects, as in the latter, articular disease often, perhaps in most cases, arises in the cancellated ends of the bones. The state of constitution, too, that gives rise to disease of the bone, is unfavorable for an operation, much more so than the worst cachexia induced by simple joint disease.

*History of a Gun-shot Wound in which the Patella was carried away, and the Knee-joint completely laid open, successfully treated by*

W. Ward, Esq., of Huntingdon.—On the 2d of November the contents of a gentleman's gun struck the patella on the outside of his knee, carrying away the whole of that bone, except a small, solid, triangular portion which still remained attached to the ligament; there was a nearly circular wound of the integuments, completely exposing the joint, and sufficiently large to admit the whole hand into the joint between the tibia and femur; but the cartilages of these bones appeared uninjured. Rest, poulticing, and the semi-flexed posture constituted the chief treatment. No unfavorable symptoms, either local or constitutional, occurred during the progress of the case, nor was the patient's pulse even in any degree accelerated. On the 21st of January following, the wound was healed, and the patient able to dress himself and sit up in a chair. In a short time, with the aid of a suitable splint and bandage, he went upon crutches; and in March he was able to ride many miles on horseback. This case confirms the observation of writers that large wounds of joints are not so commonly followed by severe constitutional disturbance as small or punctured wounds.

*Case of Dislocation of the Shoulder-joint, with Fracture of the Humerus.* By J. H. Hingeston, Esq.—The paper contains a very minute anatomical, pathological, speculative, and practical account of the above-named accident; rather more so, indeed, than to admit of an abridgment suited to our pages.

*On the Treatment of Incipient Phthisis.* By H. Marshall Hughes, m.d.—Dr. Hughes communicates in detail his mode of treating consumption, but the chief purport of the paper is to recommend the use of emetics in the early stages of certain cases of the disease. He gives an emetic about an hour before breakfast every morning, or every second, third, or fourth day, according to the strength of the patient and the character of the disease. He finds that the sulphate of zinc or ipecacuanha, in doses of twelve grains, or a combination of six grains of ipecacuanha, and two of sulphate of copper, the most desirable forms and proportions. He says their uniform effect, with a very solitary exception, has been very materially to relieve, and in not a few instances entirely to remove the cough.

*On Disorders which are variable, and on the practical Inferences which are deducible from the character of Changeableness.* By T. Wilkinson King, Esq.—This paper, to be appreciated, must be studied in the words of the author, who "considers the fact that many diseases are variable to be a law, second only in importance to the most general law that medicine embraces."

*On the Episternal Bones occasionally found in Man.* By T. W. King, Esq., (with Plates).—For an account of these bones, as given by M. Breschet, see Brit. and For Medical Review, Number XV., p. 248. In Mr. King's description we can discover nothing new.

*On some Supplementary Muscles of the Anus, described by* Dr. Horner, of Philadelphia. By T. W. King, Esq.—The muscles noticed by Dr. Horner consist of fibres better than an inch long, lying longitudinally, in bundles between the internal sphincter ani and the mucous membrane.

Mr. King, who furnishes a drawing of these muscles, considers them true retractors ani.

*Case of Transposition of the Aorta, Trachea, and Œsophagus; Tuberculated Liver, and Scirrho-cancer of the Rectum.* By Henry Ewen, Esq. (With a Plate.)—In this case the arch of the aorta ran behind both the trachea and œsophagus—between them and the vertebral column; but there was no transposition of the cavities of the heart. Four branches arose from the arch. The patient was sixty-five years of age. The other lesions had no connexion or dependence on the malposition of the great vessels.

*On the Forms of the Cartilages which keep open the principal divisions of the Bronchial Tubes.* By Jonas King, Esq.—Dr. Horner has described certain crescentic cartilages in the bronchial tubes. Mr. King, following up the subject, has here given a plate of them, together with a more minute description of their forms and uses than that given by Dr. Horner.

*Case of Urinary Calculi formed on a piece of straw.* By Henry Norris, Esq. (With a Plate).—The patient had been in the habit of relieving himself of difficulty in making water by the introduction of fine straws. The portion, the subject of the incrustation, had slipped accidentally into the bladder about a month before his death.

*On the History of a supposed Hermaphrodite.* By Robert Merry, Esq. *Its Dissection* by Sir Astley Cooper, Bart. (With Plates.)—The patient (Mary Bennett) died in her eighty-sixth year, of a gradual decay of her powers. She was very masculine in appearance, but never shaved; she led a very laborious life, and disliked the society both of men and women; she never menstruated. Summing up the abnormal anatomical circumstances of the case, Sir A. Cooper says "this woman, therefore, differed from others in the magnitude and length of the clitoris, in the absence of the external orifice of the vagina, and in the imperfect development of the ovarium."

No. XI.

*Operation for the Radical Cure of a Reducible Inguinal Hernia.* By Bransby B. Cooper, F.R.S.—The operation performed in this instance was that recommended by M. Gerdy, for the radical cure of a reducible hernia; and in its results has been satisfactory. The patient was a tall muscular man, aged twenty-two. The hernia was of seven years' standing, and had been brought on by violent exertion. At the time of the operation the rings were so open that the tumour came down even by the effort of changing position in bed, giving the sensation as if the belly was not strong enough to retain its contents. He had been subjected to a fit of strangulation shortly before, from which he was with difficulty relieved by the taxis.

"On the 10th of June, the patient was brought into the operating-theatre and laid upon a table on his back, with his chest and thighs raised for the purpose of relaxing the abdominal muscles. Mr. Cooper then commenced the operation by pushing a portion of scrotal integument before the fore-finger of his left hand through the external ring, into the inguinal canal, as high as he could pass it, and upon the finger he then introduced a director. A long needle fixed in a wooden handle, and having the eye near its point, armed with a double silk ligature, was then carried along the director to the very extremity of the invaginated skin, and was pushed through the tendon of the external oblique muscle and the skin, so as to make its appearance about an inch and a half above Poupart's ligament: one end of the silk was then retained by an assistant, and the needle drawn back again into the inguinal canal, along the other end; when it was again pushed through the abdominal parietes, in a similar manner as before, about four lines distant from the other end of the thread, including necessarily so much of the skin between the two silks, which were now tied over a piece of bougie so as to retain the invaginated portion of the skin within the inguinal canal. A piece of lint wrapped around a director, and dipped into liquor ammoniæ, was pushed into the cul-de-sac of skin thus formed, and the surface well rubbed with it, in order to remove the cuticle and promote an inflammation of the cutis, so as to obliterate this integumentary canal, and to form a plug sufficiently firm to protect the future descent of the hernia."

The application of the ammonia produced intense pain, which was but little relieved by opium, and continued more or less for two days. The surface of the skin suppurated. No symptoms of peritonitis showed themselves. 14th, the ligature was removed, as purulent discharge was most freely established, but the pressure on the part was desired to be continued. In some days farther, all uneasiness left the patient, and he felt the affected side as firm as the other, which the thickening in the course of the canal seemed to justify. In the middle of July he left the hospital and returned to his work. Early in August, however, a small portion of intestine descended while at work, in consequence of weakness of his truss, which being changed for a stronger one, he became enabled to perform all the duties of his situation. September 21, the man presented himself at the hospital to show himself to Mr. Cooper; and stated that he has been able to keep at work regularly ever since he has had the stronger truss on, and that his hernia has not again descended.

*Case of Cerebral Disturbance dependent upon Disease of the Pericardium.* By Dr. Yonge, of Plymouth.—This case is reported to corroborate some views of Dr. Bright's, published in vol. xxii. of the Medico-Chirurgical Transactions. The patient, a young man, suffered in the first instance, from rheumatic fever, which run into chorea. The convulsions at one period put on a character resembling tetanus and opisthotonos, and the distress in swallowing food or medicine was not unlike that in hydrophobia. The patient died in about six weeks from the first invasion of the illness, exhausted by want of rest, laborious spasm, and probably want of sustenance. No abnormal lesion was discoverable anywhere except in the heart, which presented marks of pericarditis, with thickening and opacity of the lining membrane of the left cavities, especially about the neighbourhood of the mitral valve. The particular view which the case is supposed to corroborate is "that the more frequent cause of chorea, in conjunction with rheumatism, is the inflammation of the pericardium; and that the irritation is communicated thence, probably, to the spine, just as the irritation from other parts."

*Observations on Diabetes; with cases illustrative of the efficacy of ammonia in the treatment of the disease.* By George H. Barlow, m.a. & l.m.—Reasoning from physiological and pathological considerations, Dr. Barlow joins in the belief that the sugar found in diabetic urine is formed in the primæ viæ at an early stage of the process of sanguification, and is not necessarily connected with a perverted action of the kidneys; and he considers the increase in the quantity of urine to be due to the diuretic action of the sugar on the kidneys. The essential character of the digestive process, in health, consists, according to Müller, in its not only effecting the solution of the food, but in its likewise annulling the peculiar properties which the nutritive matters may owe to the source whence they are derived; that is to say, in dissolving the food and converting it into albumen.

"In this disease, on the contrary," says Dr. Barlow, "the saccharine particles of the food are not changed in the stomach; whilst the starch, which most articles of vegetable diet contain in considerable quantities, not having its peculiar properties annulled, and its proneness to the saccharine fermentation being favoured by the warmth and moisture of the stomach, is converted into sugar, which, being readily soluble, is absorbed into the circulation.

"It appears, then, that, owing to a deficiency in the assimilating powers of the stomach, a lower organic product, sugar, is taken up into the system, in place of a higher organic product, albumen. This lower organic product is inadequate to perform the duties of the higher; and is, according to laws already referred to, respecting the action of the kidneys, removed from the system by these organs.

"If this view of the matter," Dr. Barlow continues, "be correct, the next enquiry is, what are the inferences to be drawn from it respecting the treatment of diabetes. The first is obviously one, the correctness of which has long been acknowledged and confirmed by experience, namely, the avoidance of all saccharine and amylaceous articles of food, the latter of which, from their tendency to saccharine fermentation, are, I believe, productive of as much mischief as the former. I have, however, little to add to what is generally received on this point, farther than to insist on the advantages of the cruciferous vegetables as articles of diet; the use of which is not only in accordance with the view taken above, but has received the sanction of many experienced physicians. Not only do greens, broccoli, turnip-tops, sea-kale, water-cresses, &c. tend to obviate the loathing which is often felt by these patients, when restricted to an animal diet, but they exert a decidedly beneficial influence over some of the symptoms.

"The next indication appears to be to introduce into the stomach a highly azotized substance, and, at the same time, by a diffusible stimulant to exalt, if possible, the assimilating powers of that organ; both of which ends appear likely to be attained by ammonia.

"Such exercise as the strength of the patient will enable him to take, and the use of a warm bath occasionally, where it can be obtained, are also valuable adjuvants. The accumulations which not unfrequently take place in the large intestines, and which are, I believe, but a consequence of the participation of the bowel in the general muscular debility, must necessarily impair these functions of the chylopoietic viscera, which it must be our main object to restore. The union of a purgative with a tonic, which rhubarb affords, seems to point it out as the most eligible remedy in these cases: this I have generally exhibited in combination with sulphate of potass; and aided its operation, if necessary, by castor oil."

The sesquicarbonate of ammonia is the preparation recommended by Dr. Barlow. He has found that under its use the function of skin is generally restored; and although he has sometimes thought that it was aided by opium in bringing about this end, yet in some cases the same effect has been produced without the use of the latter. He prescribes it in doses of from five to eight grains and upwards, with a few minims of tincture of opium, in some bitter infusion, every six hours; animal diet, with greens, being given at the same time. Dr. Barlow relates four cases, which, he thinks, show that the practice founded on the foregoing principles is by no means devoid of success; although he is far from affirming that the remedy will prove a certain cure for all the cases of this disease, which is by some considered incurable.

*Observations on Abdominal Tumours and Intumescence. Illustrated by Cases of Diseased Liver.* By Richard Bright, m.d. f.r.s.—Dr. Bright proposes in the present communication to follow out the subject of abdominal tumours, drawing the illustrations from the *liver*, as he has already done in previous memoirs, from the ovary, the kidney, and the spleen, and from the development of hydatids, (see Br. and For. Med. Review, vol. VII. p. 462.) The memoir is a very important one, and demands so full an analysis, that we shall defer the notice of it to our next Number, when we hope to be able to devote more space to it than at present.

*Report on Primary Syphilitic Cases.* By C. Aston Key, Esq.—This report is divided into two parts, of which one is given in No. X. and the other in No. XI. The whole memoir is of so valuable a character, and so full of important practical matter, that we shall here give a somewhat extended analysis of it, notwithstanding that we partially noticed it on a former occasion (vol. X. p. 381), and although we have devoted an article in the present Number to the subject of syphilis.

Out of 719 cases of men with primary venereal sores, admitted into Guy's Hospital since 1825, the following varieties presented themselves:

1. Aphthous chancre . . . . 85

2. Do. with bubo . . . . . 58

3. Do. with open bubo . . . . 41

4. Do. with phymosis . . . 123

5. Do. with phymosis and open bubo 4

6. Raised chancre of the outer prepuce, 44

7. Do. with bubo . . . . . 13

8. Raised chancre on scrotum . 4

9. Indurated chancre . . . . 23

10. Do. with bubo . . . . . 9

11. Do. with phymosis . . . . 4

12. Phymosis with chancres at extremity of prepuce . . . 46

13. Do. with bubo . . . . . 8

14. Irritable chancre of inner prepuce and glans . . . . . . 41

15. Do. with bubo . . . . . 9

16. Phagedenic sore of outer prepuce 11

17. Do. of glans and inner prepuce . 23

18. Do. with bubo . . . . . 7

19. Do. with phymosis . . . 7

20. Sloughing chancre . . . 9

21. Sloughing chancre with bubo . 3

22. Do. with phymosis . . . 17

23. Phagedenic bubo . . . . 8

24. Paraphymosis . . . . . 13

25. Do. with sloughing of prepuce and glans . . . . . 2

26. Do. with ulceration of prepuce . 15

27. Warts on glans and inner prepuce 16

— Warts, with phymosis . . 11

— Do. with phymosis and sloughing prepuce . . . . . . 2

— Warts, with paraphymosis . . 1

— Warty ulceration of anus . . 4

— Condylomatous sores about the anus and scrotum . . 12

28. Phymosis from gonorrhea . . 24

29. Suppurating bubo from do. . . 22

The nature of a chancre, Mr. Key apprehends, will depend on the depth to which the action of the poison penetrates. In the aphthous chancre, this action appears to be confined to the surface of the cutis, blistering as it were the epithelium, without indurating the surrounding tissue. Such a sore may heal quickly; but, when it is more tardy and its stages more distinct and protracted, the poison affects the parts to a greater depth, and what was at first a simple vesicle, becomes a sore with a thickened base; or it will sometimes heal, leaving behind a very slight induration, and again break out into a virulent sore, exhibiting the indurated form of venereal ulceration. Respecting the question of diversity of poisons, Mr. Key considers that it must remain in abeyance until experiments, conducted on a large scale by persons qualified for the purpose, shall have brought together an irresistible mass of evidence; and, at all events, he regards the settlement of this point one of minor importance, and considers that in the present state of our knowledge, the only well-grounded mode of proceeding is to describe and distinguish sores according to their characters, with a view of determining by experience the best mode of treatment for each. Mr. Key's own feeling in regard to this matter is that the line attempted to be drawn between the different kinds of sores is too defined, inasmuch as nature points out no such line of demarcation. Each sore is found occasionally to run imperceptibly into one of another class, so that it is difficult to decide to which it belongs.

A large number in the above catalogue of primary sores is classed under the head "aphthous." The causes of the varieties Mr. Key attempts in some measure to point out. The hardness attending a sore on the genitals, and which has been regarded by some as characteristic, cannot in itself be depended upon for determining the syphilitic or non-syphilitic nature of the case; the site of an aphthous chancre greatly modifies its condition; on the glans or inner prepuce, the sore, together with the extent of poisoned tissue, is often so superficial as to be within reach of a mild caustic, and to be therefore curable by one or two applications of the nitrate of silver; at the corona glandis, on the other hand, there is glandular structure and cellular tissue; and then we find this kind of chancre almost always firm at the base, deep in its action, and ragged instead of being smooth on its surface. On this sore the nitrate of silver has less influence, because it does not penetrate deep enough to reach the extent of the poisoned structure. Friction, scabbing, injudicious applications of nitrate of silver, long persistence of the ulcer, &c. all retard the cure and increase the tendency to hardness. Sores on the fold fringing the prepuce and occasioning phymosis are usually remarkable for their hard base, and yet, not possessing the other characters of a poisoned ulcer, yield to the simplest mercurial treatment. It would appear, then, that induration, though usually attending a chancre when seated in some tissues, cannot, when absent or present, negative or decide the action of the virus. Mr. Key remarks farther, as follows:

"Finding that chancres possessed every variety and shade of character, I came to the conclusion, either that it must be occasioned by the difference of situation which it occupied on the penis, or by a varied action or intensity of the poison, or by a peculiarity of constitution. The former position, as affording a satisfactory explanation, is wholly untenable; for though situation must be allowed to modify in some respects the appearance of sores, yet as every variety of sore occasionally appears on the same part, it is obvious that some other cause must be in operation. .......... At first it appears inconsistent with the definite progress of disease arising from one poison, that in one person a chancre should be an excavated sore, and in another an induration of tissue with scarcely a breach of surface; we see, however, in other diseases a similar variety of action from the same cause, cancer of the lower lip for example."

Mr. Key regards it as impossible, under these circumstances, to admit more than a "modification" of poison, which, he thinks, may be considered "equivalent to a difference of poison." This view of the matter differs but little from that of Mr. Carmichael. This slight difference, however, seems to Mr. Key essential to a true understanding of the matter.

In the majority of cases of common vesicular chancre, Mr. Key considers the nitrate of silver to be the best application. He says that "when used properly and under circumstances which ought not, in the eye of an ordinarily judicious practitioner, to forbid its employment, it altogether destroys the seat of the syphilitic virus, and thus prevents infection, both local and constitutional. Such an effect can only be looked for when the vesicle is seen at its commencement. If the action is not wholly arrested, an eschar is formed which serves to protect the surface and limit its extension."

Mercurial applications seem, in the early periods of such sores, to be particularly noxious, as if the action was rather increased than stayed by them; the common astringent salts, as the preparations of lead, zinc, and copper, varied as the sate of the sore will bear, check the disposition to spread, and bring on an appearance of granulation. But Mr. Key speaks of these matters to inculcate discrimination in the use of mercury, not to forbid its use. On the contrary he may rather be regarded as a mercurialist.

But though, in the aphthous sore the tendency to spread under the mercurial applications is remarkable, in the more decided chancre the disposition to be "set astray" is less and the mercurial applications will agree better. "In these sores, local mercurial action does not render the secretion copious, nor does it render the surface yellow, loose, and spongy, or the edge disposed to break up as it does on the aphthous sore; on the contrary the edge becomes less raised and firm, and not disposed to extend by ulceration; the secretion is altered but not increased, the surface becomes more solid and fibrinous and inclined to granulate. If they present not the most decided characters, but verge towards the aphthous ulcer, a combination of the two plans may be had recourse to; an astringent may be used for the purpose of protecting the sore from the injurious effects of mercurial action; thus it may be dressed with the black wash, and washed with a pretty strong solution of some of the salts alluded to. The tone and vigour of the tissue are preserved by the astringent, while the mercurial corrects the morbid action induced by the virus."

The following observations of Mr. Key are so judicious, that our readers will be obliged to us for giving them insertion in full:

"In the treatment of primary sores I commence with mercurial medicines as soon as the preceding indications show the sore needs them, and carry them to the extent that the patient's constitution is able to bear. The principles by which the practitioner should be guided, in deciding on this difficult point, are those of general pathology. The remedial agency of mercury in arresting the progress of syphilitic virus is known to all, and acknowledged by all who study the course of this disease and the action of this medicine; but the numerous conditions that interfere with its action as a remedy and tend to convert it into a poison are less known, because they are more difficult to appreciate. I know of no rules that can be laid down for the guidance of the practitioner, except such as are so general that they can hardly serve as rules; they are rather principles than rules; and where the straight line of action afforded by a rule fails, as in this, and indeed in every disease it occasionally does, principle comes to our aid as a never-failing guide."

Mr. Key describes three kinds of sores attended with induration as a distinguishing feature. The first is that known as the Hunterian chancre; even this may exist long without being followed by constitutional syphilis. Mere hardness is not alone decisive of the presence of the poison. Mr. Hunter himself did not, as has been imputed to him, measure venereal sores by the degree or extent of the induration. The second kind of indurated chancre occurs more often in private than in hospital practice. It commences with a thickening of the cutis or subjacent cellular tissue without breach of surface. If neglected, as it commonly is from the supposition of its harmless nature, these appearances increase until the skin becomes glossy, and at length excoriated. The excoriated part is highly florid and prominent, and a minute quantity of secretion can be seen oozing from it: there is not any appearance of ulceration. Such a sore, as far as Mr. Key's experience has gone, is always followed by absorption and a train of secondary symptoms. In the treatment, two circumstances force themselves upon the attention of the surgeon—the inability of the sore to bear mercurial applications, and the necessity of giving mercury in a cautious manner. Mercury should be used at once; but the form of the medicine should be such as will be least likely to increase the constitutional irritability of the patient, and effective enough to induce a curative action. The character of the secondary symptoms following this sore is usually severe even when uninfluenced by mercury. The third variety of induration resembles in many respects the foregoing. The virus neither excoriates nor ulcerates the skin; there is entire freedom from inflammation and consequent excoriation and ulceration, the action being of the most chronic kind.

"There is here no impediment to the prompt administration of mercury till the gums are affected, and keeping up the action till the mass softens and subsides. The constitution is usually in a state to bear it, and unless it is given to the full extent to produce its specific effect, secondary symptoms will arise on the skin and throat. The length of time that usually is allowed to elapse between the period of infection and the commencement of remedies, gives the opportunity of absorption; which in all the instances that I have seen of this form of the disease, has invariably occurred in a marked degree. The freedom from irritation of the part also invites the application of a mercurial; and the mercurial ointment is the best that can be used."

Although this notice of Mr. Key's paper is not designed to be more than an abstract, it may not be out of place, in a question of experience like the present, to state that we know of two well-marked cases of the latter kind of sores, which have been got to heal without mercury, and which, nevertheless, have not been followed by secondary symptoms, although many years have now elapsed since their occurrence; and this is not stated in an anti-mercurial spirit, for in both instances it was the obstinacy of the patient and not the judgment of the surgeon which saved the former from a salivation. It is the occurrence of such cases as these that embarrasses the mind and prevents it from adopting, unhesitatingly, any code of rules on the subject of syphilitic diseases.

In the management of venereal affections we should endeavour to distinguish between the essential characters of the unmixed effects of the poison and those features which they acquire from accidental circumstances. Phagedena is among the latter; and this is owing to an excess of irritability. Chancres are sometimes rendered irritable from local causes, as from improper applications, the action of the urine, the position of the sore on the frenum, &c.: this state, aggravated by improper diet, irregular hours, or other such circumstances, produces an irritability of the nervous and vascular system, which, too, is often increased by the use of mercury. Here after withdrawing mercury and restoring the tranquillity of the sore by emollients and purgatives, or sometimes by nitrate of silver, Mr. Key administers the cold infusion of sarsaparilla in lime water with the most beneficial effect. The irritable sore at the extremity of the glans usually extends as far as the influence of the urine reaches, generally to about the size of half a sovereign. It spreads slowly, making every now and then an abortive attempt to granulate, which ends in covering the surface, not with fibrine, but with a soft yellowish coating.

"Mercury, here, is rarely admissible; it is better to begin, as soon as this disposition is observed in the sore, with the application of a solution of nitrate of silver, from three to six grains to the ounce, as can be best borne; and to continue it as long as the coating of the ulcer is indisposed to become firm in texture. As soon as the whitish slough becomes firmer in consistence, it shows that the disposition to ulceration on the surface is on the decline; and in this state it will bear a weak solution of mercury, occasionally applied at first as a lotion and afterwards as a continued dressing. A grain of the bichloride dissolved in an ounce and a half of water, with the addition of three or four minims of the hydro-chloric acid, forms a good lotion; and alternated with the astringent already mentioned or with one of the metallic sulphates, will be found to correct the sore as soon as any application that can be used. Smearing the sore with oil, at each time of micturition, protects it from the contact of the urine, and is not to be neglected."

Phagedenic ulceration is a state of weakness accompanied with an excess of action in both the vascular and nervous systems of the part. The part first becomes inflamed and exceedingly painful; the inflamed tissue breaks down and ulcerates away; the former healthy limits of the sore in their turn undergo the same process. The constitutional state of the patient is evinced in his irritability of manner and loss of rest; in his pallid aspect and slightly vascular conjunctiva, while the heart acts quickly but feebly. An inflamed ulcer must be distinguished from an irritable one, as the treatment applicable to the one tends to the aggravation of the worst features of the other. The vivid colour, the fibrinous deposit, the ichorous discharge, and the thickened edge, all evince the presence of inflammation; while the absence of these signs, and in their place a degree of sensitiveness disproportioned to the extent of inflammation, or a disposition to spread by ulceration, is evidence of an irritable state of sore. But though the extreme of each class is thus distinct enough, the line that divides them is not so clear or defined; and many sores exhibit more or less of both characters.

"The degree of inflammation present and the degree of irritability existing, combined in various proportions, are the conditions that modify the progress of ulcers; and the discernment of the practitioner should be directed to ascertain in what proportions they do exist. Inflammation in one degree, combined with irritability, leads to destructive ulceration; a higher degree of inflammation with a less degree of irritability leads to the yellow slough; while the highest degree of inflammatory action with a farther diminished irritability, produces the dark slough or common gangrene."

Mercurial action is here wholly inadmissible. It tends to increase irritability, to lessen the powers of the patient, and therefore to quicken phagedenic action. Opium must be given to obtain rest. Moderate doses only are required, except in some few cases that hard drinking and debauchery have rendered uncontrollable by smaller doses of opium. When this object is obtained the sore improves. If the case be marked by both vascular and nervous excitement, the cold infusion of sarsaparilla is the remedy that deserves our confidence. When, in place of a vascular conjunctiva and flushed face and white tongue, the aspect bears marks of depression and debility, quinine or ammonia, or similar stimulants, are called for. The remedy, however, on which most reliance is to be placed is iodine and its combinations; its property seems to be stimulant or tonic; it increases vital energy and action, rendering the pulse strong and full; it improves the appetite and powers of digestion; hence its benefit seems especially adapted to that kind of ulceration which depends on want of power combined with excess of irritability. Both in primary and secondary forms of this disease it is often found to arrest the progress of syphilitic action in persons who cannot bear mercury. This latter medicine and iodine have each their respective benefits, and it may be said that iodine fulfils that in which mercury is deficient. In the normal forms of syphilis the latter is rarely found to disappoint the practitioner; and in the anormal forms of primary sore it rarely fails to do harm; while iodine exerts, comparatively, little influence on the normal chancre, chiefly confining its good services to the sores that are set astray by some peculiarity of constitution. The action of mercury is sometimes wanted for sores that, having shown an anormal disposition, have lost ground under the action of iodine, and yet will not granulate or cicatrize. The syphilitic action still predominates in them and yields only to mercury, which, then, should be given in the most guarded manner.

The second part of Mr. Key's report is principally taken up with observations on the nature and treatment of phymosis in connexion with chancre. The author enters also at length into a defence of Mr. Hunter from charges of certain erroneous views regarding syphilis attributed to him, and has come to the conclusion that the principles both of Mr. Hunter and of Mr. Carmichael are thôse by which the profession is guided in its views regarding the nature of the disease and the employment of remedies for it.

No. XII.

The first article in this number is on *Epilepsy*, from the pen of Dr. B. G. Babington; its objects are, in the words of the author, "to give some illustrations of the disease itself, and to offer some reasons for thinking that it depends on a functional not on a structural change; and some grounds for the belief that in many instances it admits of cure."

In pursuance of these objects the author proceeds briefly to notice the appearances exhibited by the disease during its attack in man and in those domestic animals which are liable to it, and to narrate some cases illustrating its principal varieties. The last of these cases is cited by the author as a striking example of the intermittent character which epilepsy occasionally exhibits. It is so remarkable a case that we shall extract it in an abbreviated form.

A married woman, æt. 50, after severe mental distress on account of the loss of her children, became subject to fits of crying in her 44th year; headaches, to which she had always been liable, soon after became frequent and violent; to these succeeded pain at the side of the neck, extending in an hour or two to the top of the head, and confining her to bed. In her 46th year she had a fit that lasted four hours and a half, and kept her in bed a fortnight; and from this time she became affected every alternate day, at first with fits of crying of six hours' duration, afterwards by a state approaching to catalepsy, and finally by a state of imbecility, during which she was incapable of making the slightest exertion. A second epileptic fit occurred at the end of two years, a third eleven months after the second; and from this time they recurred every two or three months, the attack always happening on an ill day. On the intervening days "she is as well as ever she was in her life;" and "such is the regularity," adds Dr. Babington, "with which these alternations occur, that she may be considered as having her life equally divided between sanity and imbecility."

In endeavouring to investigate the nature of epilepsy we may proceed, as the author observes, either to seek for its analogy with other physiological or morbid states of the body; to infer its nature from the constitution, age, &c. of those attacked; to trace its remote effects on the living or dead body; or, lastly, to note what kind of remedies are most useful in treating it. On each of these points the author makes some observations, but we must confine our notice to the first and last. After comparing the phenomena of epilepsy to those of nightmare, of mesmeric convulsions, and of concussion from a blow on the head, to the latter of which he considers them most analogous—

"I would wish," continues Dr. Babington, "to draw attention to an analogy which exists between the functions of the brain as affecting reason and consciousness, attributes of the sensorium, and the functions of the nerves as affecting sensation and motion. It seems to me that the same cause, whatever it be, which operates on one portion of the sensorium, so as to produce consciousness and reason, when operating on another portion of the sensorium, namely, that which is connected with the nerves of sensation and motion, produces corresponding results in the phenomena which they exhibit. Thus, I venture to think, that profound coma of the sensorium has its analogy in total paralysis of sensation and motion of the trunk and extremities; and that, in all probability, a similar cause to that which suspends reason and consciousness in the one case, suspends sense and motion in the other. Thus again, where reason and consciousness and the other attributes of the mind are not suspended, but disordered and uncontrolled; where, in other words, there exists a state of mental imbecility, insanity or idiotism—I find an analogy between this state and that of chorea, affecting the nerves of sensation and voluntary motion of the trunk and extremities. Lastly, I find an analogy between the sudden affection of the sensorium which we are now considering, namely, epilepsy, and that particular morbid affection of the nerves of sensation and motion which is productive of muscular spasm or cramp."

On the subject of treatment Dr. Babington observes:

"Where the irritation, which I suppose to be the proximate cause of epilepsy, is complicated with and dependent on organic change of structure, we cannot of course expect that treatment of whatever kind will either effect a cure or throw any light on its cause. Again: where it is combined with a state of plethora, never occurring but when the person is in a horizontal position or after a full meal, and attacking those whose habit is gross and circulation forcible, means the opposite of those which would be adopted to remove the proximate cause may be needed. Bloodletting may be necessary, as well as active evacuants, and an antiphlogistic line of treatment; but with these exceptions, it is my belief that the same class of remedies which is best calculated to give tone to the system under tic douloureux, and other spasmodic states of the nerves of motion and sensation dependent on nervous irritation, is also most applicable to this disease; and that the various preparations of bark, of iron, of arsenic, of silver, or of zinc, are those from the administration of which we shall derive the greatest advantage."

Amongst them Dr. Babington, on the whole, gives the preference to the sulphate of zinc, which, if not quite so efficacious as the nitrate of silver, is free from the objections to which the latter is subject. "Like all other remedies," he observes, "it will often fail; but whether useful or not, it has the merit of being a safe remedy, and that when continued for a longer period and in very much larger doses than is usually supposed." In some cases the author has given as much as thirty-six grains three times a day, and though these large doses are by no means always necessary, they sometimes are so. He has found this amount taken equally as well in solution as in pills, care being taken gradually to increase the dose as in the case of tartar emetic.

The second paper, by Mr. A. S. Taylor, is a *Contribution towards Determining the minimum dose of Arsenic necessary to destroy Human Life.* This is a question to which it is desirable on many accounts to obtain a correct answer, but at present no sufficient data exist on which to ground one, and accordingly authorities differ much in their estimate of the dose required to cause death.

"According to Hahnemann," observes Mr. Taylor, "two grains of arsenic might suffice to destroy life in the course of a few days, but he adduces no case in support of this opinion. Dr. Christison, in his Treatise on Poisons states, that the smallest dose of the *solid* poison which he has read of amounted to thirty grains of the powder. This dose proved fatal in six days. The smallest actually fatal dose he has found recorded is four grains and a half, but in this case the subject was four years old: death took place in six hours. In this instance the poison was taken in solution."

Dr. Lachen, of Angers,* inferred, from a case which came under his notice, that even less than two grains may prove fatal; but we agree with Mr. Taylor, that in this instance too many sources of error were present to allow of our placing much confidence in the conclusions drawn.

Mr. Taylor proceeds to relate a case of poisoning by arsenic accidentally contained in port wine, where severe vomiting, lasting three or four hours, was caused in a child æt. 16 months, a lady æt. 52, and a gentleman æt. 40, by doses of one third of a grain, one grain and a half, and two grains and a half respectively. Several days elapsed before the gentleman quite recovered, but eventually all three did well: a result, in part at least, to be attributed to the speedy rejection of the poison along with the food they had just taken.

* British and Foreign Med. Rev. Oct. 1839.

On examination it was found that not less than 28·8 grains of arsenic were dissolved in the twenty-four ounces of wine; besides which, sixty grains remained in powder at the bottom of the bottle. How the wine had become contaminated could not be ascertained, but it had been received in that state from the merchant; and in all probability, as Mr. Taylor suggests, the bottle had been formerly used to contain some arsenical preparation for destroying vermin, and had not been properly cleaned; in support of this opinion he notices several similar cases, one of which proved fatal.

The third paper by Mr. King is a continuation of one* which we noticed in a former number of this Review, *on the Safety-valve Function of the Heart*. In the next article Mr. Lever has furnished *a Statistical Report of the Guy's Hospital Lying-in Charity*, from its establishment in 1833 to 1840, during which time 4664 women have received the benefit of the charity. Mr. Lever has embodied in his report, tables showing the number of each sex born alive and those still-born; the varieties of labour which ended in still-birth; and the proportionate frequency of the various kinds of labour: for these we must refer to the original paper. He has added some remarks, in which he compares the results exhibited by these tables with those furnished by other institutions; some of these we shall notice.

Face presentations appear to have been comparatively much more frequent at Guy's than in the Dublin Lying-in Hospital; in the former they were as 1 in 179 of all cases, in the latter as 1 in 504. Merriman gives 1 in 450, Mad. Boivin 1 in 275, as the proportion. Premature birth was induced in 6 cases; in only 1 was the child born alive. Secale was but sparingly used, only once in 292 deliveries, affording a creditable contrast to the practice of those gentlemen who boast their experience of its efficacy in *hundreds* of cases. The preparation employed was the æthereal tincture, made as recommended by Dr. Rees, in the Med. Gaz., for April 4, 1840. The instrumental deliveries were 46 in number, or as 1 in 101 cases; in Dublin the proportion was 1 in 114: of these, 9 were delivered by forceps, 12 by the vectis, and in 25 cases the head was lessened. Of the 9 delivered by forceps, 5 children were still-born, and only 1 of the 12 in which the vectis was used. Thirty-three were twin cases, or 1 in 141 births, a low proportion as compared with England generally, in which twin births are as 1 to 92, or Scotland, where they are as 1 in 95, or Ireland, where the proportion is 1 in 62. Fourteen cases of placenta presentation occurred, or 1 in 333 births, a proportion four times as great as that found in the Dublin Institution, where they were as 1 in 1492; two of the cases ended fatally to the mother, and eight of the children were still-born. Forty deaths of women occurred during the seven years, or 1 in 117 cases; the great majority of these were from puerperal fever. This result must, we think, be looked on as a favorable one, especially, as the author observes, "when we take into consideration the miserable condition of many of the patients admitted to the benefits of the institution, the privations they undergo, and particularly the intemperance and imprudence in which many of them indulge." This is a valuable communication, and highly creditable to the author.

* Guy's Hospital Reports, No. iv.—Br. and For. Med. Rev. Oct. 1837.

The next is a valuable paper from the pen of Dr. Bird, *on Electricity as a remedial agent in the Treatment of Diseases.*—This is one of many remedies which has suffered from injudicious advocacy; for whilst some have attributed to it extraordinary and mysterious powers, others, failing to discover these, have decried it as unworthy of the slightest confidence; and with the majority of practitioners it has either fallen into entire disuse, or is employed only in some few cases when everything else has failed. In consequence of this the management of this remedy has been left in the hands of unprofessional persons, who, anxious to extol their specific, and incapable of distinguishing between cases likely to be benefited by it and those in which it can do no good, electrify all who come, puff the cures, and are discreetly silent about all failures. Now as we are satisfied that electricity does occasionally prove eminently serviceable, we welcome any judicious attempt to place it on its proper footing as a remedial agent. This we consider Dr. Bird's to be, who has pointed out those diseases in which it has been found most serviceable, and has related a number of cases of each treated in the hospital, sufficient, we think, to prevent the suspicion that the physician has deceived himself in estimating the effects of his remedy.

Electricity is employed at Guy's Hospital, either in the form of sparks drawn from the patient in shocks from the Leyden jar, or in galvanic shocks administered by means of an electro-magnetic apparatus.

The diseases in which it has proved most serviceable in the hands of Dr. Bird, who superintends its use in all cases treated at the hospital, are chorea, paralysis from functional disorders of the nervous system, and amenorrhœa. " A considerable number of cases of chorea," observes Dr. Bird, "have been submitted to electrical treatment since the publication of Dr. Addison's paper on the use of electricity in certain spasmodic affections, in a former number of these Reports. Of these cases notes of thirty-six have been preserved in the report-book, the histories of the others not being complete from the irregular attendance of the patients."

Of the above thirty-six cases, twenty-nine are reported to have been cured, and five relieved; one experienced no relief, and one left under alarm at the remedy. In the majority of these cases the only medicines contemporaneously administered were occasionally mild purgatives, and these as well as other medicines had often been employed before without avail. The form of electricity employed was that of sparks taken in the course of the spinal column every alternate day, for about five minutes each time, or until the appearance of a papular eruption, often excited by the remedy in this form. Several cases are detailed at length, but for these we must refer our readers to the work; the sixth affords a remarkable instance of spasmodic action of the muscles of the neck, causing involuntary movement of the head towards one side or the other with such force as to threaten strangulation, and occurring so frequently as to oblige the patient to keep his head steady whilst walking by holding his nose; in this instance electricity was of decided service.

*Chorea.* Dr. Bird "has never seen any good effect to result in cases of chorea from the transmission of electric shocks along the affected limbs: on the contrary, in every instance the involuntary movements have been increased, often to an alarming extent; and if employed when the patient was convalescent, it has invariably aggravated every symptom, and often rendered the patient as bad as when first admitted under treatment."

"*Paralysis.* Paralytic affections constitute a prominent feature in the cases which have been referred to the electrical room of the hospital; of these, forty-four cases have been fully reported by the clinical clerks, and may be found on record in the hospital books. Of these cases it may be generally remarked, that those in which the paralysis, whether of sensation or motion, or both, depended upon exposure to cold or rheumatism, upon some functional affection often of a local character, or on the impression produced by effusion in some part of the cerebro-spinal centre which had become absorbed under the influence of previous treatment, the result of the application of electricity was most successful, whilst in those cases in which the paralysis depended upon some persistent structural lesion, whether produced by accident or otherwise, I never saw the slightest benefit result."

In dropped hand from the effects of lead, electricity was employed in the form of sparks drawn from the upper part of the spine. In the majority of cases medicines were also administered, as the general health is usually deranged in this affection. Eleven cases are reported, of which five were cured, and four relieved under the use of electricity; in two cases no relief ensued.

Of rheumatic paralysis arising from exposure to damp, a current of cold air, &c. ten cases are reported, of which five were cured, three relieved, and two derived no benefit from electricity.

Of thirteen cases of paralysis from affection of the nervous centres, but where no proof existed of the presence of organic lesion, six were cured, and two were relieved under the use of electricity, four received no benefit.

Amongst the cases narrated is the following one which shows well the almost instantaneous effect occasionally resulting from the use of electricity; it would have proved quite a godsend to a lay electro-magnetist:

"Stephen Burn, aged eleven, admitted Jan. 15, 1840, into the hospital, under Dr. Addison, labouring under total loss of motion of the right leg and side, which appeared seven weeks before, without any very apparent cause, whilst in bed. He has been gradually getting worse, and has been cupped, taken mercury, had his head shaved, &c. without any marked benefit. He was carried from the ward into the electrical room being quite unable to walk. Sparks were freely drawn from the spine and affected limbs. The effect was remarkable, for the boy almost immediately recovered power over the previously paralyzed side, and he walked back into the ward with only the aid of a stick. After attending a few days longer he was presented completely well.

"*Amenorrhœa.* Scarcely any cases have been submitted to electrical treatment in which its sanatory influence has been so strongly marked as in those in which the menstrual function was deficient. . . . . . . . . . . The rule for ensuring success in the great mass of cases of amenorrhœa is sufficiently simple: improve the general health by exercise and tonics; remove the accumulations often present in the bowels by appropriate purgatives; and then a few electrical shocks, often a single one, will be sufficient to produce menstruation, and at once to restore the previously deficient function. . . . . . . . . . . The mode of applying the electric shocks in these cases is the following: Let the patient be placed on a chair or stool, press the brass knob of a director against the sacrum, and if the stays be loosened, so that only the linen intervenes between the latter and the knob, no further exposure is necessary. A second director, furnished with a chain connected with the outside of an electric jar, is passed by the female attendant under the patient's dress, and the knob is pressed against the pubes. The jar is then charged, and its ball touched by a third director connected with the one held against the sacrum by means of a chain. The shock thus passes through the patient's pelvis: and should be repeated ten or a dozen times. The jar employed should hold about a quart, and be about half charged."

In confirmation of the above observations, a table is given showing the result in twenty-four cases of amenorrhœa treated by electricity; four cases of well-marked chlorosis with an irritable state of the uterus, and in these no benefit was derived; the other twenty were free from this complication, and all were cured.

In a paper *on the Real and Supposed Pathological Conditions of the Urine*, Dr. Rees recurs to a subject treated by him formerly in a paper published in the Medical Gazette, namely, the appearance of a precipitate simulating albumen, when nitric acid is added to the urine of patients who are taking either copaiba or cubebs. As a ready mode of distinguishing between the true albuminous precipitate and these resemblances, Dr. Rees recommends the use of ferro-cyanuret of potassium as a precipitant, the urine having been first acidulated with acetic acid. Albumen if present is immediately thrown down, but no precipitate ensues where the appearance has been due to the other causes mentioned; or if the acid should have clouded the urine, this is not increased by the addition of the ferro-cyanuret.

Where heat is used as the test for albumen in urine, an error may arise from the appearance of a precipitate due to the presence of earthy phosphates. The frequency of such an occurrence is greater than might have been anticipated, amounting to not less than 7 per cent. in 482 cases taken promiscuously from the hospital wards, showing how important an error might be committed, in endeavouring to ascertain the comparative frequency of albuminous urine, if heat only were employed as the test.

It has been stated that the urine becomes albuminous during salivation, Dr. Rees shows that at least this does not always take place, since in fifteen cases in which the urine was tested during salivation, no albumen was found to exist.

In a short paper *on Stricture of the Urethra, Catheterism, and False Passage*, Mr. Cock has thrown together some cases intended as a warning to surgeons who are addicted to the heroic method of treating old strictures by what our neighbours call *catheterisme forcé.* Several of the cases afford striking illustrations of the bad results frequently attendant on this mode of treatment, and the observations which accompany them are very judicious.

*Cursory Observations on some Cerebral Affections of Children.* By H. M. Hughes, m.d.—The principal object of this paper is to state shortly some of the difficulties attendant on the treatment of the cerebral diseases of children; especially as regards the diagnosis between infantile fever or, as Dr. Hughes prefers to call, "irritative fever of children," and hydrocephalus; and between the latter complaint and the hydrencephaloid affection described by Dr. Marshall Hall.

Of the close alliance between infantile fever and hydrocephalus, and of the difficulties which not unfrequently prevent our coming to a decided opinion on the nature of the case, in the early stage at least, every practical man must, we should have thought, have been aware, had not a late writer, quoted by Dr. Hughes, asserted "that the two diseases can scarcely be confounded." Dr. Hughes thinks that in many of those cases in which hydrocephalus appears to supervene on irritative fever, the progress of the case has been really such as it appears to have been, and that complication does not always exist from the commencement of the malady, an opinion in which we agree. Nor, we may add, is hydrocephalus the only disease which may be thus excited by infantile fever. In the same way, tubercular disease in the lungs and bronchial glands of children may be developed, if it do not actually originate during the progress of infantile fever; the tubercles if previously existing, of which there is often no evidence, being at all events in a latent state, and thus the disease which begins as infantile fever may end as pulmonary consumption. The following are the symptoms by which Dr. Hughes thinks we may generally distinguish between hydrocephalus and simple irritative fever:

"In the first stage of acute hydrocephalus, there generally exist some intolerance of light and sound, contracted pupils, and wakefulness by night and by day; while in remittent fever the patient though restless at night often sleeps soundly and comfortably during the day, the pupils are rather dilated, and light and sound are not complained of. The pain of the head in the latter affection is rather a general uneasiness, giving the child an expression of heaviness and languor, and, like the febrile symptoms themselves, is distinctly remittent; in the former it is almost always referred to the forehead, and though increased in severe paroxysms, is constant. The child suffering from acute hydrocephalus lays its head on the pillow, with closed eyes, and appears unwilling to be moved, questioned, or noticed, unconsciously moves its hands up to or over its head, and often screams and starts from severe accessions of pain, while its arms or legs are affected with slight spasmodic twitchings. That affected with remittent fever, on the other hand, is usually easily and not unwillingly roused, and though fractious and petulant, has not violent fits of screaming, moves its head without inconvenience, and while awake is almost always occupied in picking its lips or nose. The bowels are sometimes constipated in both complaints; but they are more easily moved, and when moved are more easily kept in a relaxed condition, and the motions are more slimy, fetid, and dark coloured, in the simply febrile than in the inflammatory complaint. The pulse also, which in the fever is almost sharp and frequent, is in the more grave affection often sluggish, tardy, and irregular."

In the above enumeration, Dr. Hughes has omitted to notice vomiting. This symptom, though not unfrequently present in simple infantile fever, is less constant and less urgent in that disease than in the first stage of hydrocephalus. In acute hydrocephalus vomiting is one of the most frequently present of the early symptoms, and though it may last only for one day or even less, it is generally very urgent whilst it lasts, everything being rejected which the child swallows. When this symptom is present, with a belly flaccid and free from tenderness on pressure, it is, we think, one of the most characteristic that can be mentioned of incipient hydrocephalus.

In an article *On the Existence of Arsenic as a natural constituent of the Human Bones*, Dr. Rees has detailed a series of experiments upon large portions of bone earth, prepared as directed by Orfila, and tested by means of Marsh's apparatus and by sulphuretted hydrogen, which latter was ascertained to be the more delicate test of the two. By neither of these means could the slightest trace of arsenic be detected, and Dr. Rees comes to the conclusion that Orfila is mistaken in stating that this metal naturally exists in human bones, and that by some unfortunate accident it must have been present, either in his apparatus or in the materials used during his analysis.

In the next paper, Mr. Taylor, after briefly noticing those metals which chemists have succeeded in detecting in the fluids or solids of animals, to which they had been administered, relates some experiments by which he satisfied himself of the presence of lead in very minute quantity in the milk of a cow which had eaten a pot of paint containing the mineral.

Mr. Henry Oldham gives an *Account of a Fœtus in Utero invested by False Membrane*, with the view of "showing the extent to which the cutaneous surface of the embryo may be inflamed, and that the inflammatory product may be the effective cause of certain deformities of the fœtus."

"On opening the amnion a quantity of dark-coloured fluid escaped ....... Patches of a reddish-brown coloured deposit were visible on the amnion ...... The fœtus was covered throughout with a coating of a similar red-brown colour, dotted and gritty on its surface, which masked the face, so as completely to obscure the features. The limbs, thus surrounded, appeared rigid and deformed. On detaching a portion of this covering from the face, it was found to be a firm, well-formed membrane, which could be peeled off from the subjacent cutaneous surface: and when removed, the features, as the eye, nose, and mouth, were detected properly developed, the nose only being slightly flattened. This membrane was equally thick and firm throughout the body, and appeared to exert a constrictive and constraining force on several parts, producing deformities."

To the above succeed several short papers of more or less interest; amongst others *a remarkable Case of Enlargement of the Breast*, related by Dr. Ashwell, which we must, though unwillingly, pass by without further observation, and proceed briefly to notice the two remaining articles contained in this volume.

The first of these, by Dr. G. H. Barlow, is entitled *Observations on the Laws which regulate the deposition of Tubercles, with practical inferences applicable to the prophylactic Treatment of Phthisis.* In our enquiries into the laws which regulate the establishment of tubercular disease two principal questions suggest themselves: 1st. As to the causes that produce the tubercular diathesis. 2d. As to the local conditions which favour the development of tubercles in any particular organ. The latter of these Dr. Barlow proposes to consider, and in reply to it propounds as a law: "*That any organ is most liable to become the seat of tuberculous deposit when its vascular and functional activity bears the greatest ratio to the other organs of the body.*" In illustration of this law, he cites, as a well-known fact, the greater frequency of tubercles in the brains of young children, in whom that organ is relatively largest; in the abdominal viscera of older children; and in the lungs of youths at the time when these organs are attaining their full development. Again; in children, the absorbent system is in its greatest activity, whilst the genital organs are inert; in the former tubercles are very frequent, in the latter as rare.

"It is, however, in the lungs," proceeds the author, "the most frequent seat of tubercular disease, that we are to look for the full elucidation of the laws which regulate its development, and I am especially anxious to direct attention to this part of the subject, as from it arguments have been drawn which have been thought to oppose the law which I have enunciated."

We shall endeavour to give an abstract of the arguments adduced by the author.

Previously to birth, when the liver is the depuratory organ, and the function of the lungs is at zero, tubercles rarely exist in the latter organs. In childhood, though often present in the lungs, they are generally less advanced than in any other more active organs, as the brain, or mesenteric glands. In early youth, Dr. Barlow conceives that a change takes place in the proportionate activity of the liver and lungs, the latter being developed at the expense of the former. When the lungs are sound and the chest large, this goes on safely; but if otherwise, the liver retains its former proportion, and the belly is tumid and respiration impeded; or the lung is over-excited and disease occurs. Hence the necessity of attention to the prevention of phthisis at this time. In hot and moist climates, like India, the liver is active and relieves the lungs from part of their duties; whereas in temperate climates, the liver being less active, unless stimulated by animal food, the lungs are over-worked and become prone to tubercular disease. Again, as regards the part of the lungs in which tubercles are most frequent, Dr. Barlow attributes their constant presence at the summit of the lungs to the greater activity of function in this part, in which he is directly opposed to the views of Dr. Carswell. The check given to the progress of tubercles in the chest during the increased activity of the uterus in pregnancy; and the absence of tubercles from portions of lung which have been compressed in cases of pneumo-thorax, the result of phthisis, afford, in the author's opinion, further proofs of the truth of this law, as does the frequent presence of bronchitis as a precursor of tubercles in the lungs, and militate, in his opinion, against the opinion of Dr. Stokes, that atrophy of the lungs attends the early stages of tubercles.

The arguments adduced by Dr. Barlow in support of his views, and which we have very briefly and imperfectly noticed, though not conclusive, are ingenious and well worthy attentive consideration, and as such we commend them to the reader.

The last paper contains *A brief History of the last Illness of Sir A. Cooper, and of the examination after death of his body.* It is carefully and neatly drawn up, and will scarcely admit of condensation. Considerable emphysema of the lungs with enlargement of the heart were the most important morbid appearances discovered after death; Sir Astley Cooper had also directed special attention to the remains of two cured herniæ, an umbilical and a congenital inguinal, the appearances of which are well described.

